# Intelligent diagnostic model for malaria parasite detection and classification using imperative inception-based capsule neural networks

**DOI:** 10.1038/s41598-023-40317-z

**Published:** 2023-08-17

**Authors:** Golla Madhu, Ali Wagdy Mohamed, Sandeep Kautish, Mohd Asif Shah, Irfan Ali

**Affiliations:** 1 Department of Information Technology, VNR Vignana Jyothi Institute of Engineering and Technology, Hyderabad, Telangana 500090 India; 2 Operations Research Department, Faculty of Graduate Studies for Statistical Research, Cairo University, Giza, 12613 Egypt; 3 Applied Science Research Center, Applied Science Private University, Amman, Jordan; 4 LBEF Campus (Asia Pacific University of Technology & Innovation, Malaysia), Kathmandu, 44600 Nepal; 5 College of Business and Economics, Kabridahar University, Po Box 250, Kabridahar, Ethiopia; 6 School of Business, Woxsen University, Kamkole, Sadasivpet, Hyderabad, 502345 Telangana India; 7 Division of Research and Development, Lovely Professional University, Phagwara, 144001 Punjab India; 8 Department of Statistics & Operations Research, Aligarh Muslim University, Aligarh, 202002 India

**Keywords:** Diseases, Epidemiology

## Abstract

Malaria is an acute fever sickness caused by the Plasmodium parasite and spread by infected Anopheles female mosquitoes. It causes catastrophic illness if left untreated for an extended period, and delaying exact treatment might result in the development of further complications. The most prevalent method now available for detecting malaria is the microscope. Under a microscope, blood smears are typically examined for malaria diagnosis. Despite its advantages, this method is time-consuming, subjective, and requires highly skilled personnel. Therefore, an automated malaria diagnosis system is imperative for ensuring accurate and efficient treatment. This research develops an innovative approach utilizing an urgent, inception-based capsule network to distinguish parasitized and uninfected cells from microscopic images. This diagnostic model incorporates neural networks based on Inception and Imperative Capsule networks. The inception block extracts rich characteristics from images of malaria cells using a pre-trained model, such as Inception V3, which facilitates efficient representation learning. Subsequently, the dynamic imperative capsule neural network detects malaria parasites in microscopic images by classifying them into parasitized and healthy cells, enabling the detection of malaria parasites. The experiment results demonstrate a significant improvement in malaria parasite recognition. Compared to traditional manual microscopy, the proposed system is more accurate and faster. Finally, this study demonstrates the need to provide robust and efficient diagnostic solutions by leveraging state-of-the-art technologies to combat malaria.

## Introduction

Malaria is a life-threatening disease that involves the Plasmodium parasite, which poses a high death rate. It is transmitted to humans by biting an infected female mosquito with the parasite. Malaria is predominantly a tropical disease since mosquitoes thrive in tropical areas, and it is both preventable and treated. According to the latest Global Malaria Report, there are projected to be around 241 million malaria cases and 627 thousand fatalities worldwide by 2022^1^. Moreover, research by the World Health Organization (WHO) suggests that concerns related to COVID-19 could triple the number of malaria cases^2,3^. In response to this global epidemic, the WHO has enacted policies to prevent, treat, eradicate, and monitor malaria^4^. Malaria, a preventable disease, can be controlled and prevented if adequate processes and protocols are used, including early diagnosis of the malarial parasite^4^. Several laboratory techniques, including polymerase chain reaction (PCR), microscopy, and rapid diagnostic test (RDT) are commonly used for investigating malaria using thick or thin blood smears^5–8^. However, conventional methods tend to rely heavily on manually examining blood smears under a microscope. These methods are time-consuming, subjective, and require highly trained personnel. Additionally, the reliance on clinical experts raises concerns about the consistency and accuracy of the diagnosis. To address these deficiencies, computer-aided diagnostic (CAD) methods for malaria evaluation are being developed to reduce mortality rate^9^. Therefore, automated and accurate diagnostic systems are needed to improve malaria detection. Artificial intelligence has gained more and more attention in the scientific community. It has contributed to improving detection through various diagnostic processes. Most medical imaging analyses now incorporate CAD procedures that leverage deep learning techniques for effective model learning.

However, despite advancements, malaria remains endemic in some areas where the disease is common. Early screening plays a crucial role in detecting malaria and saving lives. Consequently, this motivates us to create faster and more accurate malaria diagnosis procedures. Recently, deep learning architectures have received much attention in terms of research and are the most important method to detect disease automatically and more accurately. These generic deep networks have played a vital role in image classification, detection, and recognition^10,11^. In a similar vein, data-driven deep learning (DL) algorithms have surpassed manually constructed feature extraction techniques^12^. A convolutional neural network (CNN) is a type of deep learning model that employs different mechanisms, such as local receptive fields, shared weights, and clustering layers, to leverage information. Its purpose is not limited to extracting features but also extends to generating predictive targets and furnishing actionable predictive models that can effectively aid physicians^10,13^. Deep neural networks have shown outstanding performance in computer vision tasks in recent years. This is done using methods like the ResNet-32 network model to identify ductal carcinomas^14^ precisely. Despite their effectiveness, CNN suffers from limitations in the modeling of spatial relationships and the lack of an internal representation of the geometrical restrictions on the image data. When these flaws are applied to microscopic cell images, the diagnostic model may be misclassified. The need for a more precise and efficient model arises to improve the performance of detecting and classifying malaria parasites. These challenges have prompted us to develop a rapid and more accurate diagnosis procedure for malaria. The specific hypotheses tested in this study include:

### Hypothesis 1

Using the inception neural network will enable the extraction of rich and discriminative features from microscopic images of malaria cells, improving parasite detection and classification accuracy.

### Hypothesis 2

The incorporation of the imperative capsule neural network will enhance the modeling of spatial relationships within the images, allowing for a more precise classification of malaria parasites.

By testing these hypotheses, the study aims to demonstrate the superiority of the proposed approach over traditional manual microscopy and other existing methods for malaria diagnosis.

This paper is organized as follows: The relevant research is presented in Section “Related works”, and the proposed inception-based imperative capsule neural network is discussed in Section “Materials and methods”. Part “Experimental results” summarizes and describes the outcomes of this network. Part “Conclusions” concludes with the article's conclusions and suggested recommendations for further study.

## Related works

Several researchers have demonstrated promising results in medical applications by using data-driven machine learning (ML) and deep learning (DL) models. This study examines contemporary deep-learning applications that elicit key decision-making factors in the diagnosis process. Liang et al.^15^ presented a 16-layer CNN to classify the parasitized and uninfected cells in thin blood smears. Features are extracted using a pre-trained AlexNet^16^, and a support vector machine (SVM) is trained on these features, and the model has an average accuracy of 97.37%. However, the transfer learning method achieves only 91.99% accuracy. Bibin et al.^17^ proposed and tested a six-layer deep belief network to detect malaria parasites in cell images. Based on their findings, the study achieved 96.4% classification accuracy on a custom dataset using training or test randomization. Dong et al.^18^ presented SVM and CNN-based approaches for classifying malaria parasites from cell images. This study attained an accuracy of more than 95% using pre-trained deep learning models such as those used in LeNet^19^, AlexNet^16^, and GoogLeNet^20^. Rajaraman et al.^21^ proposed a deep-learning model for malaria parasite detection and classification. The method visualizes the activation maps of each layer and understands the probabilities of the different layers to understand the modeling process. As a result, it obtains an accuracy of 98.61%. Mahdi Postchi et al.^22^ surveyed the latest advancements in image analysis and machine-learning techniques for diagnosing malaria through microscopy. Although many machine learning models using traditional features have been developed for image classification and decision-making, these models may lack generalization ability. Sivaramakrishnan et al.^23^ suggested a customized CNN model and evaluated the effectiveness of pre-trained and deep-learning CNN models as feature extractors for microscopic images to differentiate between healthy and parasitic blood cells. The model uses surface features to achieve more outstanding results than deep features and applies a level-set-based algorithm to detect and segment red blood cells. This model achieved 98.6% (cell-level) accuracy. Yang et al.^24^ presented a fivefold cross-validation for two-step CNN models. In the first step, the model uses an intensity-based iterative Global Mini-mum Screening method to recognize parasites, and then a CNN uses a custom CNN to classify the presence of parasites. The success rate of this method is 93.46%. Vijayalakshmi et al.^25^ presented a transfer learning method with a classification accuracy of 93.13% to discriminate between illustrations of malaria-diseased cells and healthy using the VGG16 model and a support vector machine. Madhu et al.^26^ proposed an improved dynamic routing process to classify malaria-infected cells from healthy cells using a fully trained capsule network, and the model achieved an accuracy of 98.82%. Loddo et al.^27^ used the DenseNet-201 neural network to categorize Plasmodium falciparum life stages into four groups and used two different datasets to assess the robustness of the model. The binary classification accuracy rate was 97.68%, and the multi-classification accuracy rate was 99.40%. Meng et al.^28^ proposed a neighborhood correlation graph convolutional network to identify multistage malaria parasites. The model has excellent recognition ability for multistage malaria parasites, outperforming the comparison method by at least 8.67%. Madhu et al.^29^ proposed an automated diagnostic model based on deep Siamese capsule arrays for uniquely detecting and classifying malaria parasites. When simplified on the largest test sample (test = 40%), the model achieved an accuracy of 96.61% and 98%, respectively. Ha et al.^30^ presented a semi-supervised graph learning framework to solve the problem of identifying apicomplexan parasites. Hybrid graph learning is also used in this approach to explore the relationships between different parasites with and without labels.

In malaria, the Plasmodium parasite causes an acute fever that is carried by female Anopheles mosquitoes. It produces life-threatening sickness if left untreated for a long time, and delaying exact treatment might lead to the development of additional comorbidities. A microscope is currently the most prevalent method for detecting malaria. Consequently, an automated approach to diagnosing malaria is required. This study proposes the development of an urgent, inception-based capsule network for classifying parasitized and uninfected cells from micrographs. These diagnostic models contain neural networks based on the Inception and Imperative Capsule architectures. Using a trained model, such as Inception V3, the first block collects rich characteristics from images of malaria cells. In the second block, a dynamic imperative capsule neural network classifies malaria cells into infected and uninfected red blood cells. The experiment's findings indicate a considerable improvement in recognizing malaria parasites, which contributes to better illness diagnosis and prevention.

By observing the existing challenges, this study aims to develop an automatic diagnostic prototype for classifying malaria parasites from microscopic cell images using the Inception neural network with the Imperative Capsule neural network. The preliminary results of this study are presented as follows:

- To develop an innovative approach employing an urgent, inception-based capsule network to recognize parasitized and uninfected cells from microscopic images.
- The Inception block extracts rich features from malaria cell images using a pre-trained model, such as Inception V3, which facilitates efficient representation learning to recognize the parasites.
- The dynamic imperative capsule neural network is utilized to classify microscopic images into parasitized and healthy cells, enabling the detection of malaria parasites.
- To compute routing by agreement among low-level and higher-level capsules that can be used to predict malaria cells and classify them into parasitized and uninfected cells using L2-Norm.
- This study underscores the importance of leveraging state-of-the-art technologies to combat malaria by providing a robust and efficient diagnostic solution.

## Materials and methods

### Dataset collection

**Figure 1:**
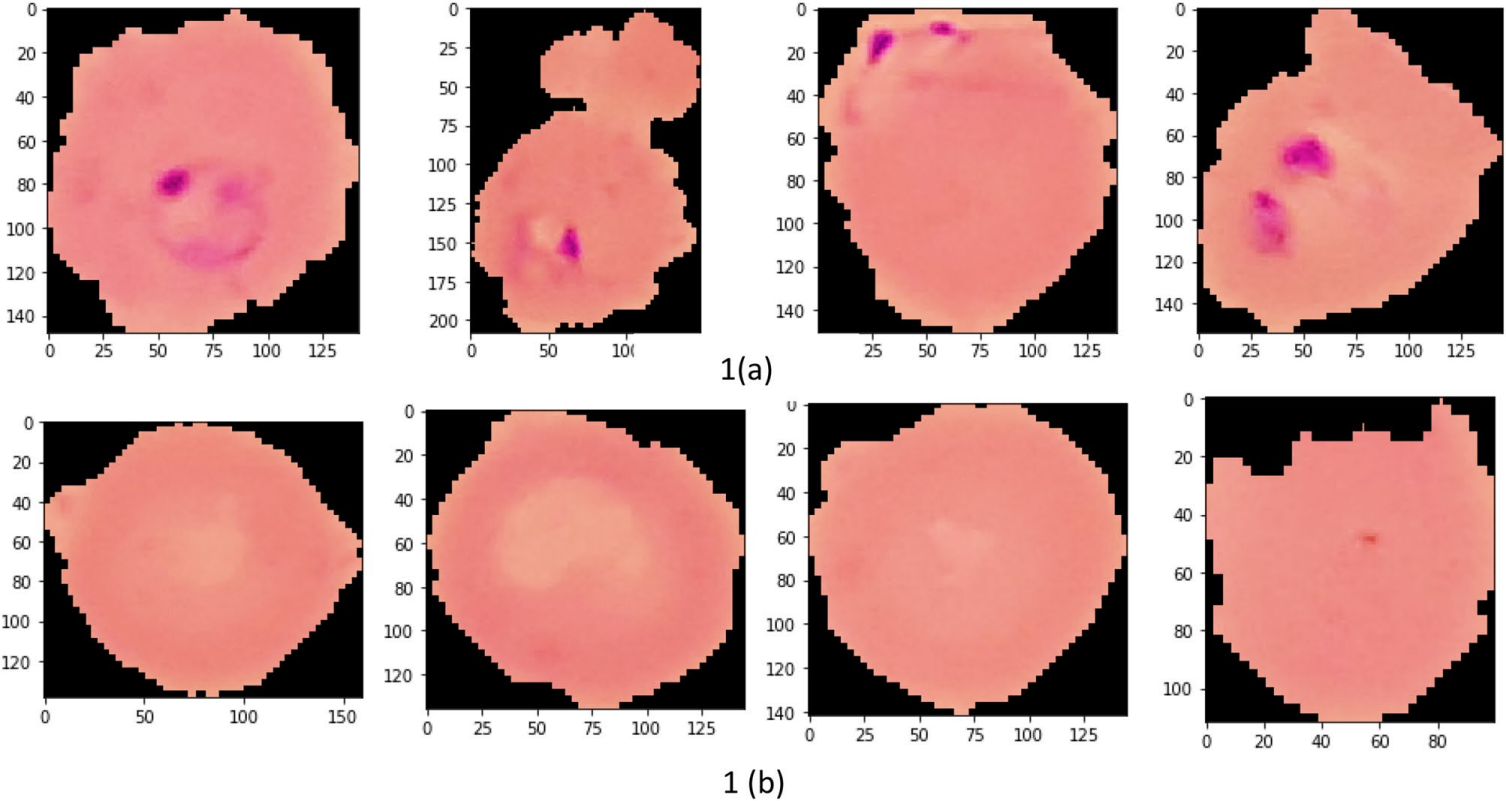
Illustration of sample malaria cell images: (**a**) Infected images; (**b**) Uninfected images (without parasites).

Images of thin blood smears containing two distinct strains of malaria—one infected and the other not—were used in the study. These samples were gathered from patients and healthy controls who had Plasmodium falciparum infections, and they were stored at the National Institutes of Health (NIH) repository, which is open to the public for study^23^. The collection includes 13,779 images of parasites and 13,779 images of uninfected cells, totaling 27,558 images of labeled and segmented cells from thin Giemsa-stained blood smear slides. Figure 1 offers some parasitic and uninfected cell images to visualize their physical traits.

### k-fold cross-validation (CV) test

The dataset contains 27,558 blood cell images with malaria-positive and negative samples, which were evaluated in our study for data sample training and testing, and used k-folds (k = 10, 20, 30, 40, 50) Cross-validation to evaluate the proposed model. As shown in Table 1, the dataset is split into training and testing subsets.

**Table 1:** The dataset distribution in training and testing with different splits.

Dataset splits	Training dataset		Testing dataset	
	Split ratio (%)	No. of images	Split (%)	No. of images
1	90	24,802	10	2756
2	80	22,046	20	5512
3	70	19,290	30	8268
4	60	16,534	40	11,024
5	50	13,779	50	13,779

### Inception neural network and the imperative capsule neural network

Geoffrey Hinton et al.^31^ motivated this research by addressing the limitations of traditional CNNs by proposing inception-based capsule neural networks, which require small data but have higher computational complexity.

**Figure 2:**
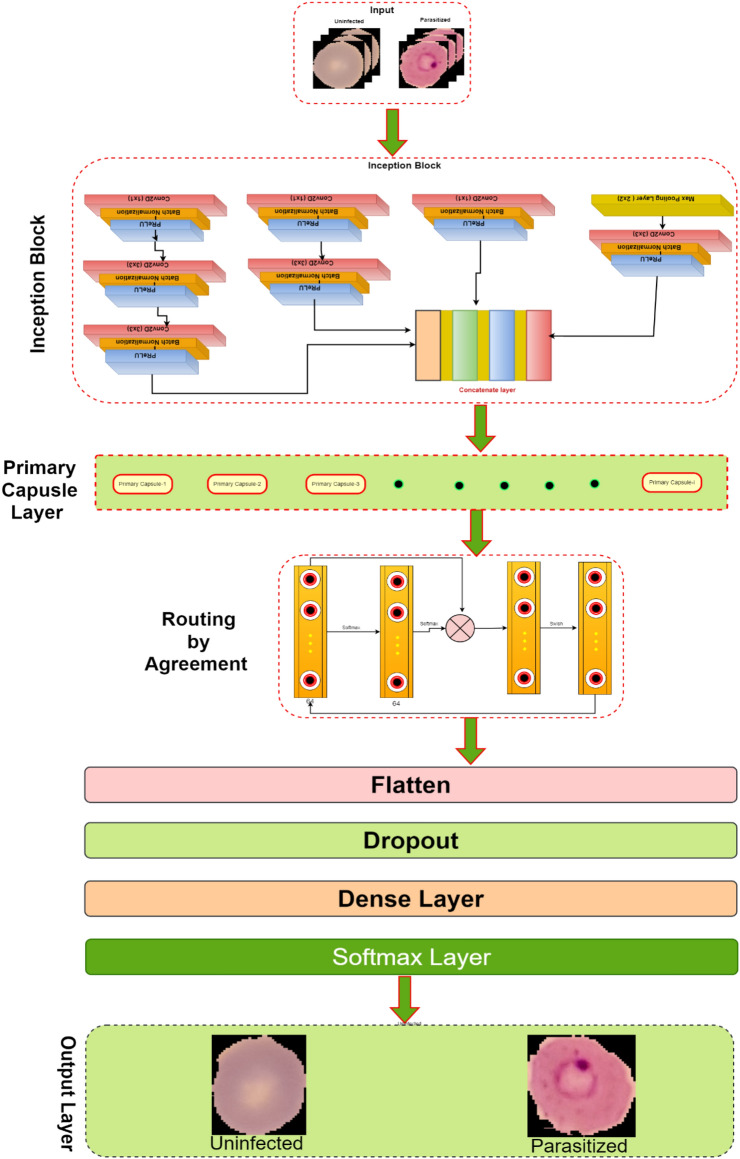
The proposed architecture of Inception-based capsule neural network.

This research develops an inception-based imperative capsule neural network for malaria detection, and its basic architecture is shown in Fig. 2, which is similar to the architecture advocated for image classification problems by Sabour et al.^31^. According to Fig. 2, input is first routed through fully connected inception blocks, which receive the parasitized and uninfected portions of the cell images as input and extract features on the parasitized and uninfected portions of the cell images. The inception block's output is used as the primary capsule layer's input. The primary and higher capsule layers utilize an imperative routing mechanism to learn the captured features by discerning the spatial orientation of the parasites on the extracted features. After multiple iterations, the resulting output is a feature vector with a length equivalent to the probability of the interval [0, 1], which preserves the object's pose information, minimizing the information loss caused by the feature vector extraction. This feature vector is then used to classify a test sample as infected or healthy cells, aiding in its classification.

### Inception neural network block

In 2015, Google introduced a module for GoogleNet^32^, also known as Inception V3, a convolutional neural network that helps us with image analysis and object detection.

**Figure 3:**
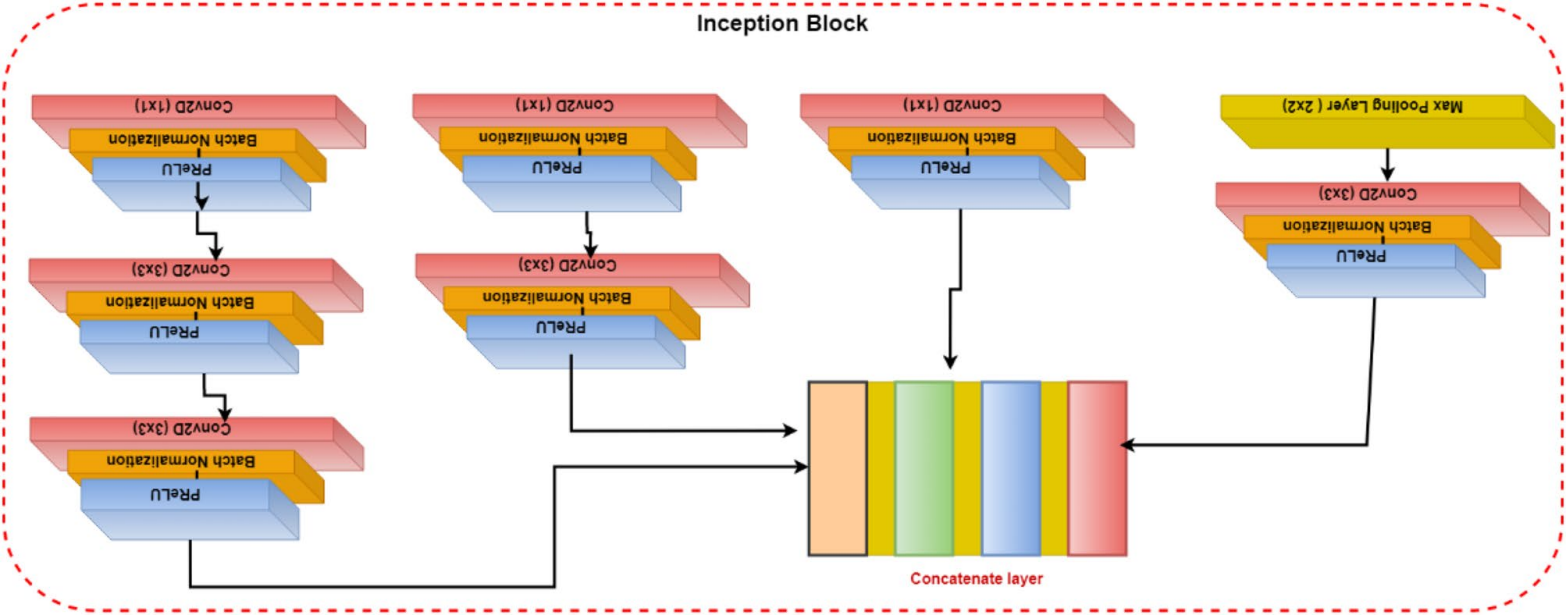
Illustration of the inception block.

Convolutional layers are frequently employed in convolutional neural networks (CNNs) to extract information from images of malaria blood cells. The CNN's initialization block, which is made up of parallel convolutional layers with filters and kernels of various sizes, extracts feature from various scales to obtain multi-view information on parasites and healthy cells. The structure of the inception block, which is used to extract characteristics at various scales, is shown in Fig. 3. To extract features at various sizes, this block has four parallel convolutional layers with various kernels (1 × 1, 3 × 3, and 3 × 3). A max-pooling layer with a kernel size of 2 × 2, a convolution layer with a kernel size of 1 × 1, and a batch normalizing layer make up the final parallel convolutional layer. Each parallel layer's computational cost and channel count can be decreased by using a 1 × 1 convolutional layer, and the model's computational cost can be decreased by employing a 3 × 3 max-pooling layer. The output feature maps of each of the four simultaneous convolutional layers are combined after computation to produce new feature maps that are used as the input for the capsule network.

### Capsule networks block

To classify the items in the MNIST dataset, Sabour et al.^31^ presented a capsule network (CapsNet). It uses a neural network to produce an output vector that includes both a scalar and a vector encoding the features of the objects in the image. In our experiment, these capsule networks are trained by carefully adjusting the number of rounds in the dynamic routing algorithm. Using Parametric ReLU (PReLU), it is possible to investigate the behavior of nonlinear activations during dynamic routing^33^. The presence of features in the form of vectors containing low-level entity instantiation parameters is estimated using the principal capsule layer. CapsNet transforms the scalar output using feature detectors in this layer, then passes the vector output of the capsules to the following layer using a modified routing method^31^. Because parameter tuning is critical for better network learning and faster convergence, proper initialization is used to start the routing procedure with kernel initializer before the primary capsule layer; the dynamic routing algorithm is activated with Glorot-normalization^34^. Each capsule, $i$ has an activity vector ${u}_{i}\in R$ in the layer of $l,$ which captures information about the features extracted from an entity (i.e., blood cell image). The output of the activity vector ${u}_{i}$ of the $i$th level capsule is fed as data into the next level layer, i.e., $l+1$ layer. The ${j}{\text{th}}$ layer capsules of layer $l+1$ will get data from ${u}_{i}$ and compute the product weight matrix ${W}_{ij}^{T}$. The results are stored in the form of ${\widehat{u}}_{(j|i)}.$ This vector is the layer of capsules $i$ at level $l$ layer, which is the transformation of the entity represented by capsule $j$ at the level of $l+1$. Then apply the transformation matrix ${W}_{ij}^{T}$ to capsule output ${u}_{i}$ of the previous layer, as shown in Eq. (1).

1 $${\widehat{u}}_{(j|i)}={W}_{ij}^{T}*{u}_{i}$$

In Eq. (1), capsule $i$ is the primary capsule layer, $j$ is the higher-level capsule layer, and ${u}_{i}$ is the output of the capsule network of the upper layer and ${W}_{ij}^{T}$ is the learnable weighted matrix between the ${i}{\text{th}}$ capsule to ${j}{\text{th}}$ capsule. Which is multiplied by each output vector and the coupling coefficient ${C}_{ij}$ is added to the linear sum stage. Then the capsules are in the higher level, which is filled with the sum of the output vector in the lower-level layer, and we add it with a coupling coefficient ${C}_{ij}$ which is computed during the routing method shown in Eq. (2).

2 $${C}_{ij}=\frac{\mathrm{exp}({a}_{ij})}{\sum_{k}\mathrm{exp}({a}_{ik})}$$

In dynamic routing, the coupling coefficient is determined by Eq. (2). In the process of calculating ${S}_{j}$ in forward propagation, ${W}_{ij}^{T}$ is set to a random value, ${a}_{ij}$ is initialized to zero, ${u}_{i}$ is the output of the previous layer, and then compute a weighted sum ${S}_{j}$ with weights ${C}_{ij}$ (the sum of these coefficients is equal to one) and it is denoted as follows:

3 $${S}_{j}=\sum {\widehat{u}}_{(j|i)}* {C}_{ij}$$

The squashing function map of ${S}_{j}$ yields the output vector ${v}_{j},$ which is obtained is defined as follows:

4 $${v}_{j}=\frac{1}{\Vert {s}_{j}\Vert \left(1+{\Vert {s}_{j}\Vert }^{-2}\right)}$$

The squashing function, defined by Eq. (4), ensures that short vectors are reduced to fewer dimensions near zero while long vectors are scaled to unit length, thus introducing nonlinearity to the capsule network. The total input Sj processed by the jth dimensional capsule array contributes to the coupling coefficient Cij. An activation function PReLU is applied to update the coupling coefficients, instead of the squashing function, by operating on Sj. During the iterative learning phase, these coupling coefficients are updated using Eq. (5), which proceeds as follows:

5 $${a}_{ij}\leftarrow {a}_{ij}+{\widehat{u}}_{(j|i)}{v}_{j}$$

In Eq. (5), ${a}_{ij}$ is a parameter used as a weighted proxy, which means that it gives higher weights to appropriate predictions, and it starts at zero and is modified as the training progress.

**Figure 4:**
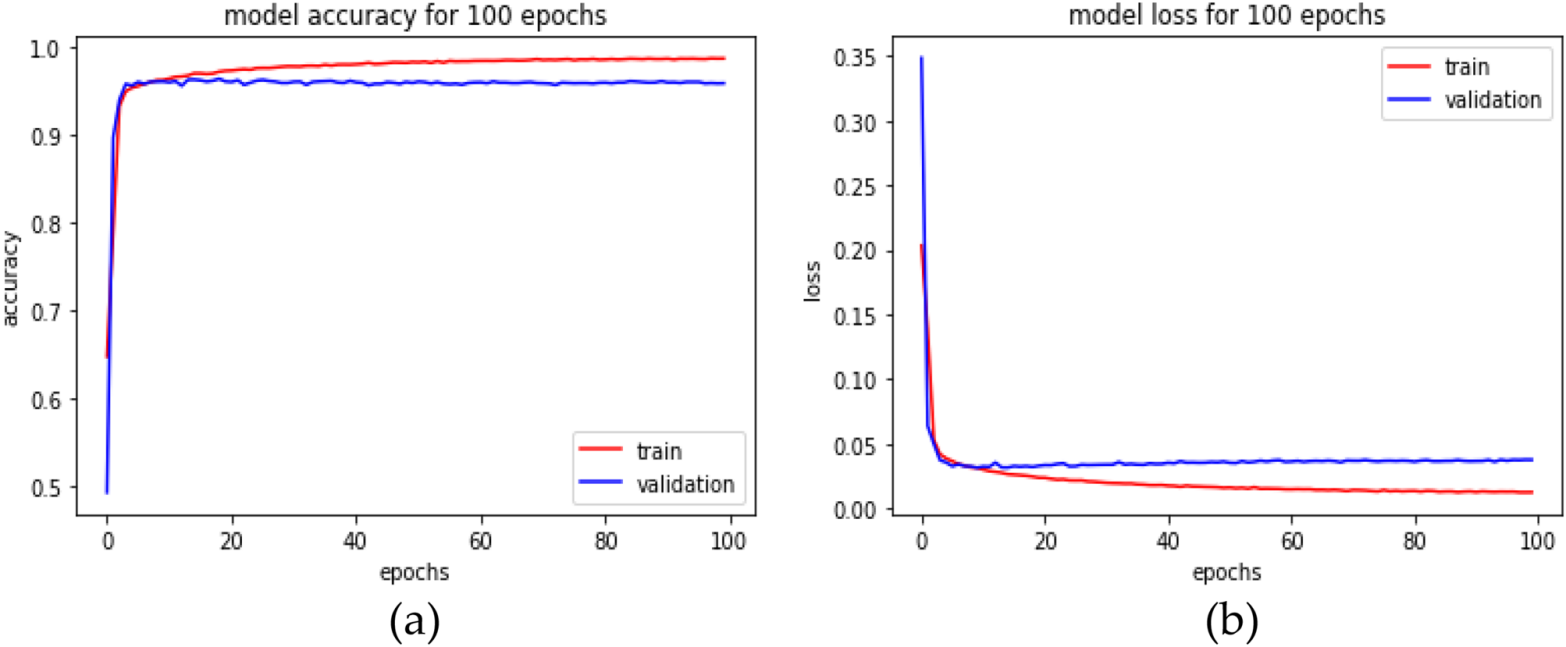
An inception-based capsule network with a router in 3 iterations, depicted as (**a**) accuracy curves and (**b**) loss decay curves.

However, it is initialized with the current input weights to improve the learning method by reducing the computational cost and improving the predictive ability. The number of routing iterations (n = 3) is used as a hyperparameter allowing one to choose a specific number of iterations during the training (here, epochs = 100) period, and the details of this network parameters are shown in Table 2. The learning period is evaluated by evaluating the convergence, and our model is repeated for only three iterations. Figure 4 depicts the comprehensive learning curves for iterations over 100 epochs.

**Table 2:** Parameters of the models used in the evaluation.

Parameters	Networks	
	Inception-v3	Capsule network
Depth	48	8
Input size	128 × 128	128 × 128
Optimizer	RMSProb	Adam
Loss function	Cross-entropy	MSE
Batch size	64	20
Learning rate	0.0001	0.007
Momentum	0.9	0.8
Gradient	L2 norm	L2 norm

*Adam* Adaptive momentum estimation, *MSE* mean squared error.

PReLU activations are utilized during the routing by agreement process to improve the understanding of feature invariance in the captured images of malaria cells. In a conventional capsule network, the squash activation function is typically used as a non-linearity. However, using PReLU as a non-linearity is believed to lead to better generalization and convergence over time. The last layer of the network comprises two capsules (parasitized and uninfected cells) reflecting the probability of the interval [0, 1] and the position information of the object, preserving the pose information to reduce information loss caused by the extracted feature vector. This enables the classification of test samples into either parasitized or uninfected cells, thus aiding in cell feeding.

### Loss function

Our current loss function^31^ also includes the mean squared error rate (MSE) alongside the marginal loss. Change the settings for faster convergence and add proper model regularization and noise addition when training the classification model with a value set to 0.45.

6 $${Loss}_{x}={T}_{x}*PReLU({m}^{+}-{\Vert {v}_{x}\Vert }^{2}+\sigma *\left(1-{T}_{x}\right)*PReLU {\left(\Vert {v}_{x}\Vert -{m}^{-}\right)}^{2}+\sum {\Vert {T}_{x}-{v}_{x}\Vert }^{2}$$

In Eq. (6), ${m}^{+}$ and ${m}^{-}$ are the category prediction values, $\sigma$ is the balance coefficient, ${T}_{x} \mathrm{is \, the \, label \, of \, category},$ and classification probability vector $\Vert {v}_{x}\Vert$ is the size. For this study, the default values are set as ${m}^{+}=0.85 \& {m}^{-}=0.15$, $\sigma =0.45$. The total loss function, in this case, refers to the loss of capsules representing both malaria-parasitized and uninfected classes.

## Experimental results

This section describes the proposed model's implementation in-depth and thoroughly analyses how well it performs under various restrictions. The proposed network was evaluated against front-line classification models created by several authors, which were pre-trained using NIH malaria datasets^23^ and other private datasets to assess whether red blood cells are parasitized or not. According to Table 3, the proposed model for malaria parasite identification and classification performed well on the NIH malaria dataset, along with the comparison findings. It is important to note that most models typically exhibit low performance on this dataset. Although their weights can handle common classification datasets, they frequently fall short because of ineffective feature extraction brought on by too much depth. Instead, the Inception-based capsule network model classifies parasitized and uninfected cells accurately during the diagnostic process by utilizing external knowledge to produce rich characteristics. On international benchmarks, the suggested model performs noticeably better.

**Table 3:** The performance of the proposed method is compared to that of other methods.

Authors	Dataset	Accuracy	Sensitivity	Specificity
Das et al.^35^	Private	0.9320	0.9410	0.8790
Dong et al.^17^	Private	0.9812	0.9732	0.9870
Diaz et al.^36^	Private	0.8500	0.9400	0.9870
Gopakumar et al.^37^	Private	0.9770	0.9810	0.9720
G.Madhu et al.^26^	NIH	0.9882	0.9836	0.9930
Liang et al.^15^	NIH	0.9730	0.9690	0.9780
Rajaraman et al.^23^	NIH	0.9860	0.9810	0.9920
Rahman et al.^38^	NIH	0.9770	0.9740	0.9790
Proposed method	NIH	0.9935	0.9957	0.9912

As stated in the Table 4, our model is assessed for layer-wise testing cell images, varying from training to 80% and testing to 20%.

**Table 4:** The performance of an inception-based capsule neural network on variant generalization tests ranges from 10 to 50%.

Split ratio	Accuracy	Sensitivity	Specificity	AUC-ROC
90% vs 10%	0.9928	0.9898	0.9899	0.9943
80% vs 20%	0.9935	0.9957	0.9912	0.9973
70% vs 30%	0.9894	0.9847	0.9890	0.9894
60% vs 40%	0.9849	0.9837	0.9912	0.9950
50% vs 50%	0.9810	0.9805	0.9858	0.9709

In this analysis, experiments are conducted on various distributions, and the suggested network's implementation, as shown in Table 4, achieves an accuracy of 99.35% and an AUC score of at least 99.73% at a test ratio of 20%. Table 4 shows the models' overall generality as measured by various standard classification metrics, including accuracy score, AUC–ROC, sensitivity, and specificity. Limiting diagnostic power does not assess the likelihood that a certain patient will acquire a disease, but it does affect diagnostic accuracy, even though they choose sensitivity and specificity. Table 5 displays the effectiveness of the suggested capsule array at various nonlinearity levels. Compared to the performance of cutting-edge pre-trained models, the generalization distribution for the training and test samples is 80% to 20%.

**Table 5:** Illustrates the performance of the proposed model compared to existing pre-trained state-of-the-art architectures.

Models	Accuracy	Sensitivity	Specificity	AUC-ROC	F1-score
VGG16	0.9603	0.9567	0.9640	0.9920	0.9560
VGG19	0.9597	0.9560	0.9632	0.9910	0.9550
Inception V3	0.9280	0.9250	0.9302	0.9760	0.9251
ResNet50 V2	0.9390	0.9356	0.9408	0.9410	0.9820
Xception	0.9470	0.9420	0.9480	0.9792	0.9439
DenseNet121	0.9562	0.9482	0.9650	0.9901	0.9480
MobileNetV2	0.9483	0.9420	0.9552	0.9880	0.9478
Capsule net	0.9518	0.9500	0.9514	0.9900	0.9498
Proposed	0.9935	0.9957	0.9912	0.9973	0.9936

**Figure 5:**
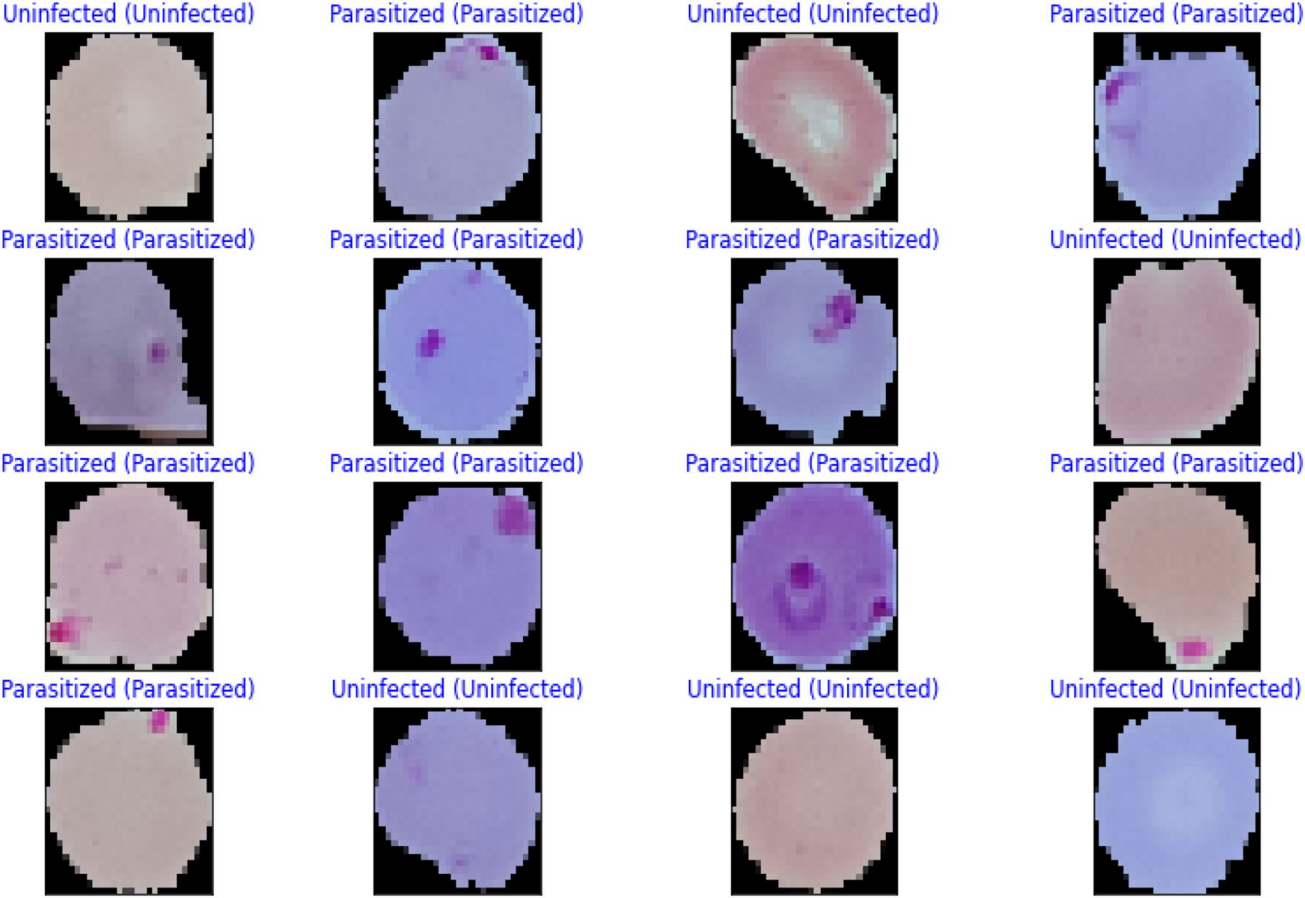
Illustration of some prediction results of the proposed model.

The performance metrics for every deep learning architecture are compiled in Table 5. The proposed malaria detection algorithm outperforms the compared deep learning models in terms of performance. The results showed an accuracy of more than 99.35%, an AUC score of 99.73%, and an F1 score of 99.36%. The accuracy score is a well-known metric with a domain that is invariant to general utility; hence it is imperative to note. As a result, the effectiveness of the suggested model is assessed using various measuring techniques. The model was created to be assessed by segregating partition samples that vary from 10 to 50%, ensuring that the model is adequately generalized. Figure 5 displays the predicted results of the suggested model on images of malarial cells. The true value is shown on the x-axis, and the model forecast is shown on the y-axis.

### Time complexity analysis

According to our study, the learning model was trained for 100 epochs to assess the time complexity of the model. The results show that our model takes around 33.8667 min for training and 3 s for complete testing, which is less than all the compared models. This study addresses the urgent need for automated malaria detection and classification. It proposes a novel approach based on integrating inception and imperative capsule neural networks. This research has the potential to significantly improve malaria diagnosis, contributing to more effective disease management and prevention. Additionally, the study contributes to the growing field of deep learning in medical image analysis. It showcases the applicability of advanced neural network architectures to address critical healthcare challenges.

## Conclusions

This research develops a deep-learning approach by combining the imperative capsule neural network with the inception neural network to distinguish between malaria-parasitized and uninfected cells. This enhances the classification accuracy of identifying malaria parasites from photographs of blood cells. With well-chosen parameters, the capsule model can efficiently finish the procedure for classifying uninfected cells or parasites into different categories. Models with different loss parameters are compared to the proposed model, and the results show that the model's performance can be increased by adjusting the loss parameters. The proposed network achieves higher classification accuracy while analyzing blood cell images for malaria than competing deep learning methods. Under the worst-case scenario (50/50 split), the model obtains an accuracy of 98.10% on the test, while on the 20% split, it achieves an accuracy of 99.355%. These experimental results are helpful since the developed model is robust and flexible and has outperformed competing models. In the work's future scope, the model may be utilized to recognize parasite species and stages in thin blood smears. This research opens opportunities for future advancements in malaria diagnosis and surveillance, including using mobile and portable imaging devices for point-of-care testing.

## Data Availability

The data that support the findings of this study are openly available in the National Library of Medicine (NLM)—Malaria Data: https://lhncbc.nlm.nih.gov/LHC-research/LHC-projects/image-processing/malaria-datasheet.html and reference number Ref.^23^.
